# Modeling the Synchronization of Multimodal Perceptions as a Basis for the Emergence of Deterministic Behaviors

**DOI:** 10.3389/fnbot.2020.570358

**Published:** 2020-12-03

**Authors:** Pierre Bonzon

**Affiliations:** Department of Information Systems, Faculty of Economics, University of Lausanne, Lausanne, Switzerland

**Keywords:** developmental cognition, behavioral learning, synchronized perceptions, neural circuit, virtual machine

## Abstract

Living organisms have either innate or acquired mechanisms for reacting to percepts with an appropriate behavior e.g., by escaping from the source of a perception detected as threat, or conversely by approaching a target perceived as potential food. In the case of artifacts, such capabilities must be built in through either wired connections or software. The problem addressed here is to define a neural basis for such behaviors to be possibly learned by bio-inspired artifacts. Toward this end, a thought experiment involving an autonomous vehicle is first simulated as a random search. The stochastic decision tree that drives this behavior is then transformed into a plastic neuronal circuit. This leads the vehicle to adopt a deterministic behavior by learning and applying a causality rule just as a conscious human driver would do. From there, a principle of using synchronized multimodal perceptions in association with the Hebb principle of wiring together neuronal cells is induced. This overall framework is implemented as a virtual machine i.e., a concept widely used in software engineering. It is argued that such an interface situated at a meso-scale level between abstracted micro-circuits representing synaptic plasticity, on one hand, and that of the emergence of behaviors, on the other, allows for a strict delineation of successive levels of complexity. More specifically, isolating levels allows for simulating yet unknown processes of cognition independently of their underlying neurological grounding.

## Introduction

Living organisms use either innate or acquired (i.e., learned) mechanisms for reacting to percepts with an appropriate behavior e.g., by escaping from the source of a perception detected as threat (even an amoeba reacts to turning away from light), or conversely by approaching a target perceived as potential food. In the case of learned behaviors, seminal work in comparative zoology has stressed the importance of unimodal percepts in the development of animal cognition leading to deterministic behaviors (see e.g., Zentall et al., 1981). The term “modality” is used here to distinguish between percepts and/or actions that can be compared and eventually equated (e.g., the color of objects to choose from) from those which cannot (e.g., a right/left spatial percept cannot be directly associated with the selection of a forward/backward gear). By definition, a deterministic behavior does not depend on randomness i.e., it follows a specific pattern (e.g., could be described by a rule). In bio-inspired artifacts (e.g., robots), such capabilities can be either built in through wired connections, as in Braitenberg (1986) vehicles, or defined in software. In this second case, a further distinction must be made as to whether a priori defined associations between modalities must be specified, or if one wants to construct a machine with evolving capabilities, i.e., encompassing learning and awareness capabilities. The problem addressed in this study is to define a possible neural basis for artifacts of this later type.

According to Pepperberg and Lynn (2000), the first level of animal awareness corresponds to the ability to follow a simple rule involving the perception of a specific item or event and then its acceptation or its rejection e.g., a case of *matching/oddity* to sample (Zentall et al., 1981; Katz et al., 2008). Whereas this first level does not allow for an immediate transfer to a similar task, an organism with the second level is aware enough of a rule to transfer it across situations and thus to adopt for example a *win/stay lose/shift* rule (Cole et al., 1982). The third level of animal awareness provides an organism with the additional capacity to refer to some previous experiences and integrate two sets of information in order for example to make a categorical judgment e.g., to *sort* items (Savage-Rumbaugh et al., 1980). These three levels thus offer a model for a developmental process based on a single perceptive modality e.g., vision. More precisely, the evolution that underlies this taxonomy shows a progressive shift from a single event involving two unimodal percepts (e.g., matching the color of two objects), toward the combination of two events involving a single percept (e.g., the view of an object that in turns triggers an episodic memory). Moreover, these successive cognitive abilities refer to static associations (i.e., they do not include an explicit time dimension, whereas later levels do) that are perceived between objects and/or events. As such, they are often said to belong to the restricted domain of *perceptual* (or access) consciousness (Block, 1995).

The introduction of a time dimension projects into the domain of dynamical systems e.g., *monitoring* (or behavioral) consciousness, in which associations between percepts are replaced by causality rules (Freeman, 1999) that can involve contextual and/or multimodal perceptions. The complexity of the corresponding neural phenomena has so far prevented the definition of models directly relating single neuron dynamics to global brain states (see e.g., Goldman et al., 2019). As an example, whereas neurological measurements (Tomov et al., 2018) have validated a computational Bayesian model positing a dedicated neural mechanism for causal learning (Gershman, 2017), nothing is known about the corresponding basic neuronal processes that could be reproduced in a bio-inspired robot.

As pointed by many authors (e.g., Carandini, 2012; Cooper and Peebles, 2015; Love, 2015), bridging the gap between brain measurements and cognitive processes requires formal models that do provide links between neural circuits and behaviors. In other words, and in contrast to formalisms that use statistical methods to search for patterns existing in a brain, one needs to consider *processes* and not just *data*. Toward this end, a new computational approach implementing a symbolic model of asynchronous neural dynamics has been proposed (Bonzon, 2017a). While this does not provide evidence about the links between neural circuits and behaviors, it allows for constructing brain structures that might be associated with higher level cognitive capabilities, stressing thus the fact that the corresponding grounded neural processes are yet unknown. Given under the form of a *virtual machine*, this framework offers an interface situated at a meso-scale level between abstracted micro-circuits representing synaptic plasticity (this representation relying on a detector of coincidence, as evidenced by neurological findings), on one hand, and behaviors, on the other; this interface thus allows for a delineation and implementation of *successive levels* of complexity.

This formalism has been used to simulate the first three levels of animal awareness and to model its possible roots (Bonzon, 2017a, 2019). In order to go beyond behaviors based on a single modality, we design and simulate here a thought experiment involving an autonomous vehicle with two perceptive modalities. Its behavior is first implemented as a random search. The stochastic decision tree that drives it is then transformed into a plastic neuronal circuit learning an appropriate deterministic behavior. This leads to posit a model of causal learning based on synchronized multimodal percepts. Extending the Hebb principle of wiring together neuronal cells (Hebb, 1949), which was originally expressed at the level of coincidentally firing neuronal cells, this new learning principle relies on the concept of *asynchronous threads* within a given *stream* (i.e., at a meso-scale level superimposed on the micro-scale level of neuronal cells). An application of this principle will reveal how inhibition/disinhibition processes of neural assemblies (i.e., a key mechanism for circuit learning as evidenced by neurological findings) allow for learning the equivalence relations among different modalities, which in turn leads to the learning of behaviors.

## Theoretical Review

This theoretical review is intended to situate our work within the overall framework of developmental cognition, and in particular to highlight the relation between a multimodal brain and synchronized processes.

### The Development of Rodent's Brain

The development of both animal and human brains includes processes ranging from gene expression to environmental inputs. It is out of the scope of this study to review all these processes (see e.g., Stiles and Jernigan, 2010). We shall rather restrict ourselves to issues directly related to our work i.e., the origin of a rodent's brain ability to represent space and the role played in this context by the synchrony of circuits.

Following the early seminal work performed on rats by O'Keefe and Dostrovsky, 1971, followed later by that of Moser and Moser (2008), which earned them together the Nobel prize in 2014, events associated with space are represented in the brain by assemblies of a variety of specialized neurons (i.e., place, head direction, grid, and border cells), which together constitute visual *receptive fields*. These assemblies form a dynamic representation of positions when one moves through the environment. This raises in turn the following question: is this ability to situate oneself in space acquired before birth or learned through encounters with the environment? Both (Langston et al., 2010; Wills et al., 2010) report that components of the brain's spatial representation systems are already present when an animal starts to move in its environment. Moreover, the evolution of this representation follows from an increase in network *synchrony* among cortex cells (Langston et al., 2010). As a result of experienced stimuli from environmental inputs, Hebbian learning eventually allows for “overwriting the earlier ante-natal configuration” (Wright and Bourke, 2013).

### Perception Development

Two successive and somehow intervened processes must be distinguished: first, *sensation*, which is the process of receiving information from the world, and then *perception*, which refers to the interpretation of that information and its contribution, via motor responses, to the choice of actions. Whereas the reception of visual information, as sketched above in the case of rodents, is achieved through receptive fields, its interpretation, which in turn is grounded in the brain, results from yet mostly unknown circuits and mechanisms. Furthermore, because of the existence of multiple sensory asynchronous organs and inputs, resulting sensations, perceptions, and actions are multimodal in nature (Bertenthal, 1996), and their proper discrimination and integration rely, among others things, on a differentiation based on a common *synchrony* (Bahrick, 2004).

### Development of Consciousness

A recurring debate about the functioning of the brain concerns the characteristics and the roles played both at the neurological and cognitive levels by *synchronous* vs. *asynchronous* processes, their relation to *conscious* vs. *unconscious* behaviors, and a possible fundamental *duality* in neural dynamics. While the synchronous activation of brain processes is widely used for describing the functioning of the cortex (Singer, 1993), diverging views apply to the specialized tasks supported by these synchronized processes. Experimental results have revealed in particular the existence of transient long-range phase synchronization leading to the hypothesis that synchronization vs. desynchronization is a candidate mechanism for controlling visual attention (Gross, 2004). Other studies related to the integration of attributes in a visual scene suggest that there is no central neural clock involved in this mechanism, thus making the brain a massively *asynchronous* organ (Zeki, 2015). In support of this diversity, results from a large scale simulation (Markram et al., 2015) report “a spectrum of network states with a sharp transition from synchronous to asynchronous activity.”

Unconscious and conscious behaviors have been described respectively as lacking conscious attention and enjoying an introspective reporting capability (Shanahan, 2009). Various studies have focused on the search for the neural activity that differentiates between the two, but their overall results appear inconsistent (Dehaene and Changeux, 2011). As an example, experiments related to a delayed matching to sample task (Dehaene et al., 2003) have suggested that the neural signature of unconscious vs. conscious perception could be a *local coordination* vs. a *global synchronization* of neural activity. Further results (Dehaene et al., 2006; Melloni et al., 2007) about the same task have concluded that *transient synchronization* is the critical event that triggers an access to consciousness. While no definite links between neural activity and conscious behavior (which would constitute *neural correlates of consciousness*) have been identified yet, it is nevertheless common to postulate the existence of a dynamical *stream of consciousness* mediated by a global workspace (Baars, 1988) defined as a distributed brain state connected to various brain areas, thus making perceptual information available to different tasks. In one of these theories (Dehaene and Naccache, 2001) pertaining to the particular case of conscious perception (referred to also as *access consciousness*), sensory stimuli are associated with a population of excitatory neurons that in turn inhibits other neural assemblies, thus preventing the conscious processing of other stimuli.

More generally, an emergent picture of the brain shows opposing spiking patterns in populations of neurons engaged in a competition (Zagha et al., 2015). The demonstration of temporal competition in eligibility traces for long term potentiation and depreciation (*ltp/ltd*) designates these traces as plausible synaptic substrate for reward-based learning (He et al., 2015). Together, these findings enforce a fundamental principle in circuit neuroscience according to which, as result of synaptic plasticity, inhibition in neuronal networks allows in turn for disinhibition and stands as a key mechanism for circuit plasticity, learning, and memory retrieval (Letzkus et al., 2015).

### The Bayesian Coding Hypothesis

According to the so-called “*Bayesian coding hypothesis*” (see e.g., Doya, 2007), the brain represents sensory information under the form of probability distributions. Statistical methods based on Bayes' theorem allow then to update these probabilities by using newly available data. Briefly stated, Bayes' theorem allows for calculating the *a posteriori* conditional probability of an event based on *a priori* existing data about both the event itself and some conditions related to that event. In other words, the brain supposedly encodes a model of the world and makes predictions about its future sensations. Predictions are compared with actual inputs and the differences between them i.e., the prediction errors, are propagated in the model (Knill and Pouget, 2004). Driven by a hierarchical structure, prediction errors from a lower-level are given as inputs to a higher-level. In parallel, feedback from the higher level provides prior data to lower levels. Let us just mention in passing that in this view, “neuropsychological deficits can be thought of as false inferences that arise due to aberrant prior beliefs” (Parr et al., 2018). However, as already noted in the introduction, the neural grounding of bayesian processes is still unknown, and a subject of ongoing controversies.

### Focus of This Work

As suggested throughout this theoretical review, associating various perceptions and/or actions through synchronization processes could constitute the basis on which to build a functional model of a multimodal brain. Somehow tautological, this idea is developed below.

Let us first recall the distinction between *sensations* i.e., the capture of sensory inputs through neuronal *receptive fields*, on one hand, and *perceptions* i.e., their interpretation through higher level neurological structures and mechanisms related to cognition, on the other. The choice of a *virtual machine* situated at an abstracted meso-scale level allows for simulating these yet unknown processes of cognition independently of their neurological grounding. Therefore, and contrary to the usual (but also controversial, because to date they do not offer any link to implement cognitive processes) approaches (e.g., Markram et al., 2015),

- individual neurons will not be simulated in any of their anatomical and/or functional details, and considered solely as communicating entities receiving and transmitting signals from abstracted aggregated sensory inputs represented by *symbolic data*

Furthermore, in order to allow for simple abstracted sensory inputs (i.e., thus ignoring their capture through multiple receptive fields),

- space will be restricted and defined as a one *dimensional axis*.

The viability of this approach has been tested in a simulated unimodal context (Bonzon, 2019) that reproduced experimental results in the field of comparative zoology. In the absence of published material matching multimodal concepts as introduced in the present study, we shall rely here on a *thought experiment*.

## Materials and Methods

The formalism and tools used in this study have been previously published (Bonzon, 2017a). In order to help the reader follow and understand the results that will be presented in the next section, we introduce them here by providing first their methodological background. This is followed in turn by an overview of these tools and some implementation details of the basic concepts they are based on.

### Methodological Background

In order to characterize different approaches in *model-based cognitive neuroscience* (Palmeri et al., 2017), Turner et al. (2017) consider a set of *neural data* denoted by ***N*** and a set of *behavioral data* denoted by ***B***, and distinguish three ways these two domains can interact i.e.,

1. using the neural data to constrain a behavioral model
2. using the behavioral model to predict neural data
3. modeling both neural and behavioral data simultaneously.

Whereas the first two cases use unidirectional statistical influence, the third one relies on a bidirectional link between measures of different modes to formalize a connection between ***N*** and ***B*** through a cognitive model. As an example of this third case, and in order to relate the model parameters that respectively predicts ***N*** and ***B***, (Turner, 2015) uses a hierarchical Bayesian structure connecting the neural and behavioral levels. All these approaches rely on statistical methods to relate data to patterns of neural activity. In order to introduce processes, as argued in the Introduction, a new type of interface borrowed from the field of software engineering is proposed under the form of a virtual machine. This new approach falls thus into the domain of *computational cognitive neuroscience* (Ashby and Helie, 2011; Kriegeskorte and Douglas, 2018).

Generally speaking, a virtual machine is a software construction having its own execution language ***L*** that emulates the execution of a program written in another higher level language ***S***, thus allowing for interfacing two domains. A classical example is given by the Java machine, where the languages ***L*** and ***S*** correspond respectively to Java *byte code* obtained from the compilation of Java *source code*. The virtual machine that we shall consider here allows for interpreting code given under the form of *logical implications* ***l*** ϵ ***L*** compiled from symbolic expressions ***s*** ϵ ***S***. With regard to the statistical framework considered by Turner et al. (2017) relating neural data ***N*** to behavioral data ***B***, we have the following bottom up correspondence:

***N* →** ***L***

***B* →** ***S***

where logical implications ***l*** ϵ ***L*** are used to deduce *virtual machine instructions* (i.e., the model's grounding) and symbolic expressions ***s*** ϵ ***S*** represent *virtual circuits* driving behaviors.

### Tools Overview

An experimental platform for a new type of brain modeling based on a virtual machine (i.e., similar to a Java machine that allows for software developments without having to worry about the idiosyncrasies of the underlying hardware), has been developed (see Bonzon, 2017a). It is defined, and thus at the same time implemented, by a logic program of about 300 lines that can run on any PC equipped with a Prolog compiler.

In a first approximation, this machine does function as non deterministic learning automaton that is defined by a repeated *sense-react* cycle of embodied cognition. In this particular instance of embodied cognition, brain processes are first abstracted through virtual *microcircuits* representing synaptic plasticity. Sets of microcircuits can be then assembled into *meso-scale* virtual circuits linking perceptions and actions.

In order to get an intuitive idea of the functioning of this virtual machine, let *Model* designate its current state, comprising various machine registers and a repository of contextual implications compiled from the symbolic expressions representing virtual circuits. At the top level, the virtual machine is defined by a *run* procedure that consists of a *loop* whose cycle comprises a *sense* procedure followed by a *react* procedure:


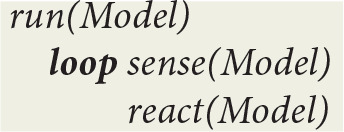


At the next level, the *sense* procedure monitors spike trains directed to sensory neurons. After capturing an *Input* interrupt, it updates *Model* registers through a transition function *input*:


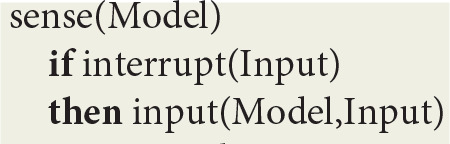


The *react* procedure in turn consists of a loop using implications in *Model* to first deduce a virtual machine *Instruction* and then update *Model* using a transition function *output*:


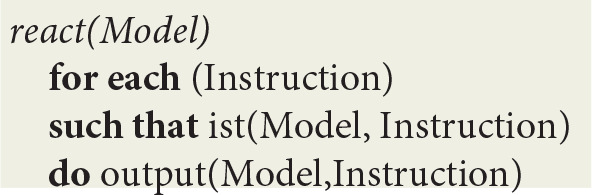


The *ist* predicate (standing for “is true”) implements contextual deduction (Bonzon et al., 2000). The *output* function corresponds to the execution of a virtual machine instruction.

### Extended Virtual Machine Definition

The virtual machine used in this study, originally designed to execute a “*sense-act*” cycle of embodied cognition as sketched above, is extended here to implement a “*sense-act-reflect*” cycle.

The basic units of processing making up microcircuits are constituted by *threads*. In computer science, a *thread* is a piece of program made of a sequence of instructions that executes concurrently with other threads to constitute a formal *process*. In the present context, a thread is similarly defined by a *tree* (i.e., a hierarchical data structure) of instructions enclosed in a *symbolic expression* that stands in one to one correspondence with a *circuit* (see section Basic Concepts Implementation). Threads are communicating entities intended to represent either a single or a group of neurons. Contrary to traditional neuron models in which their inputs are summed up, threads do process their inputs individually, thus making them a kind of free quasi-particle (Frégnac, 2017). In order implement a detector of coincidence for handling messages (Buzsaki and Llinas, 2017), synaptic plasticity is abstracted through *asynchronous communication* protocols.

This overall approach can be then summarized as follows:

- *micro-scale* virtual circuits implementing synaptic plasticity through asynchronous communicating threads are first defined
- *meso-scale* virtual circuits corresponding to basic, but yet unknown cognitive processes, are then composed out of these micro-scale circuits
- virtual circuits, represented by symbolic expressions, are finally compiled into *contextual implications* allowing for the deduction of *virtual instructions* to be eventually interpreted by a *virtual machine*.

In order to represent neurons as a kind of free quasi-particle that, according to the tri-level framework based on synaptic plasticity considered by (Frégnac, 2017), participate in multiple functional sub-networks, disjoint sets of threads form *fibers*. Fibers correspond to the formal notion of independent *processes* made of concurrent threads, and are used to model neural assemblies (Huyck and Passmore, 2013). An active thread within a fiber gives rise to a *stream*. Each communication taking place within a given stream do involve a pair of threads and entails on one side the signal transmitted by a pre-synaptic *source* thread, and on the other side its reception, via a given synapse, by a postsynaptic *recipient* thread. Similarly to a neuron, a thread can be both a source and a recipient and functions as a gate receiving incoming signals from different sources and sending an outgoing signal to possibly many recipients. There are however two essential differences between threads and neurons that allow for a single thread to represent a group of neurons i.e.,

- contrary to a neuron that alternates roles in cycles, a thread can be simultaneously a source and a recipient by maintaining parallel communications
- contrary to traditional neuron models in which incoming signals are summed in some way into an integrated value, thread inputs can be processed individually.

**Figure 1:**
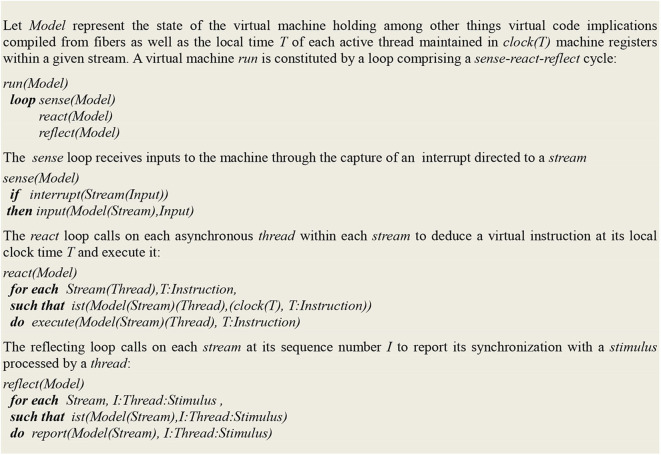
High level definition of a virtual machine run.

On this basis, the extended virtual machine is defined in Figure 1.

One must clearly distinguish here between two things i.e., the way the virtual machine is implemented, one hand, and what this machine does (say as opposed to solving differential equations in the case of a traditional brain simulator), on the other:

1. the virtual machine itself is a program, written in Prolog, which gets compiled in C and is finally executed by the native code of any computer (exactly like a traditional brain simulator)
2. this virtual machine implements a “sense-act-reflect” cycle that allows for tracing down the sequences of synchronized events associating a thread and a stimulus.

Let *Model* represents the state of the machine holding, among other things, virtual code logical implications compiled from fibers as well as the local time *T* of each thread within a given stream, which is maintained in *clock(T)* machine registers. As defined in Figure 1,

- the *sense* loop allows for the input of data from sensors
- the *react* loop, in which the *ist* predicate stands for “is true,” implements contextual deduction (Bonzon et al., 2000); more precisely, local times *T* are used to deduce and execute the next instruction; whenever an instruction succeeds, its thread clock is advanced and the next instruction is deduced, and whenever it fails, it is executed again until it eventually succeeds
- the *reflect* loop tracks down and reports synchronized events, which are defined by the association of a *stream* sequence number *I*, a *thread* and a *stimulus*.

This machine therefore essentially functions like a *theorem prover* that first deduces “just in time” virtual machine instructions and then executes them. By relying on the implementation of synaptic plasticity included in the machine state under the form of primitive built-in threads common to each stream, the execution of virtual instructions leads to a wiring/unwiring process that produces model configurations that are akin to plastic brain states. As postulated for instance by Zeki (2015), there is no central clock, thus “making of the brain a massively asynchronous organ.” There are two time scales i.e., one defined at the micro-scale level, and the other at the meso-scale level. The micro-scale time corresponds to the set of local times *T* of asynchronous threads maintained in the registers *clock(T)*; the meso-scale time corresponds to the set of sequence numbers *I* of streams maintained in registers *seq(I)*. Both of these time scales are not predefined and unfold as the model execution proceeds: more precisely, a local time *T* is incremented by the virtual machine after the successful execution of a virtual machine execution (which can be delayed, as stated above); a sequence number *I*, which corresponds to a global time within a given stream, is incremented after a move.

### Basic Concepts Implementation

As introduced above, the tools used in this study rely on three fundamental concepts i.e., the formal notions of

- a *thread* i.e., an object in context defined by a symbolic expression enclosing an instruction tree and representing a circuit
- *concurrent communicating threads* that obey various communication protocols and model a network of interactive circuits
- a *virtual machine* interpreting virtual code deduced from contextual implications compiled themselves from symbolic expressions representing circuits.

The complete formal specifications and implementation details of this formalism, which includes both the language of the symbolic expressions and that of the virtual machine instructions compiled from these expressions, can be found in (Bonzon, 2017a). The virtual machine language refers to entities such as data registers, signal queues and a content accessible memory; the *graphical representation of circuits*, which stands in one to one correspondence with symbolic expressions, is privileged here because of its more synthetic and intuitive format.

The interaction of threads obeys various communication protocols. These protocols are implemented by means of procedures that operate in pairs and are depicted in the graphical representation of circuits by symbols i.e.,

- the symbol **->=>-** depicts a *synaptic transmission* implemented by a **send/receive** pair
- the symbol **/|\** depicts the modulation of a synapse; depending of its associated thread, it corresponds to either a processes of *long term potentiation* **(ltp)** or *depression* **(ltd)** both implemented by a **join/merge** pair.

**Figure 2:**
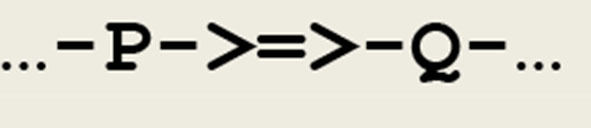
Circuit fragment implementing a synaptic transmission. A signal is transferred between **P** and **Q**, and **->=>-** depicts a synapse.

As a first example, a simple *asynchronous communication* between any two threads **P** and **Q** can be represented by the circuit fragment given in Figure 2.

**Figure 3:**
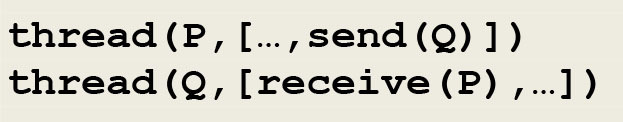
Thread patterns for a synaptic transmission. Thread **P** will fire in reaction to the capture of an external stimulus, with the **send** procedure corresponding to sending a signal, or spike train, carried by a pre-synaptic neuron's axon. In the thread **Q**, the **receive** procedure represents the reception of this signal by a post-synaptic neuron.

This circuit fragment can be represented by the two symbolic expressions in Figure 3.

**Figure 4:**
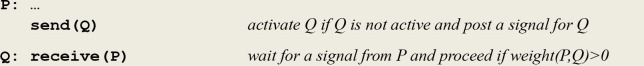
Communication protocol for an asynchronous communication. The **send/receive** protocol corresponds to an *asynchronous* communication subject to a threshold. It involves a predefined weight between the sender **P** and the receiver **Q.** This weight can be incremented/decremented by an **ltp/ltd** thread (see below). After firing thread **Q** and sending it a signal, thread **P** goes on executing its next instruction. On the other side, thread **Q** waits for the reception of a signal from thread **P** and proceeds only if the weight between **P** and **Q** stands above a given threshold.

These two expressions will be compiled into virtual code ***l*** **ϵ** ***L***, in this case logical implications implementing the communication protocol given in Figure 4.

At a meso-scale level, threads represent clusters of connected neurons i.e., neural assemblies, and the *send/receive* instruction pair is used to implement an aggregated communication mechanism, yet to be identified in actual neuronal structures. Altogether, this mechanism constitutes an implementation of synaptic plasticity in neural circuits.

#### Example: A Model of a Simple Case of Classical Conditioning

**Figure 5:**
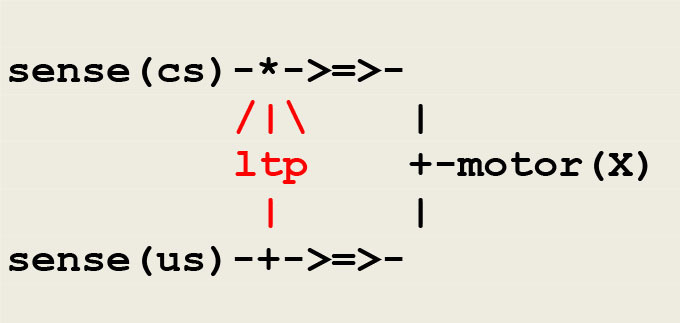
A circuit implementing classical conditioning. The symbol /|\ represents the modulation of a synaptic transmission, the sign * used in the upper branch indicates the conjunction of converging signals, and the sign + either the splitting of a diverging signal, as used in the lower branch, or a choice between converging signals, as used in the right branch instantiating the thread **motor(X)**, where **X** is a variable parameter to be instantiated into either **cs** or **us**. The thread ltp (standing for *long term potentiation*) acts as a facilitatory interneuron reinforcing the pathway between **sense(cs)** and **motor(cs)**.

As example, let us consider classical conditioning. In one experiment (Kandel and Tauc, 1965), a light tactile conditioned stimulus **cs** elicits a weak defensive reflex, and a strong noxious unconditioned stimulus **us** produces a massive withdrawal reflex. After a few pairings of **cs** and **us**, where **cs** slightly precedes **us**, **cs** alone triggers a significantly enhanced withdrawal reflex. The corresponding circuit, adapted from a similar schema (Carew et al., 1981), is represented in Figure 5.

Classical conditioning then follows from the application of hebbian learning (Hebb, 1949) i.e., “neurons that fire together wire together.” Though it is admitted today that classical conditioning in *aplysia* is mediated by multiple neuronal mechanisms including a postsynaptic retroaction on a presynaptic site (Antonov et al., 2003), the important issue is that this activity depends on the temporal pairing of the conditioned and unconditioned stimuli, which leads to implement the thread **ltp** as a *detector of coincidence* as done in the protocol given in Figure 5.

**Figure 6:**
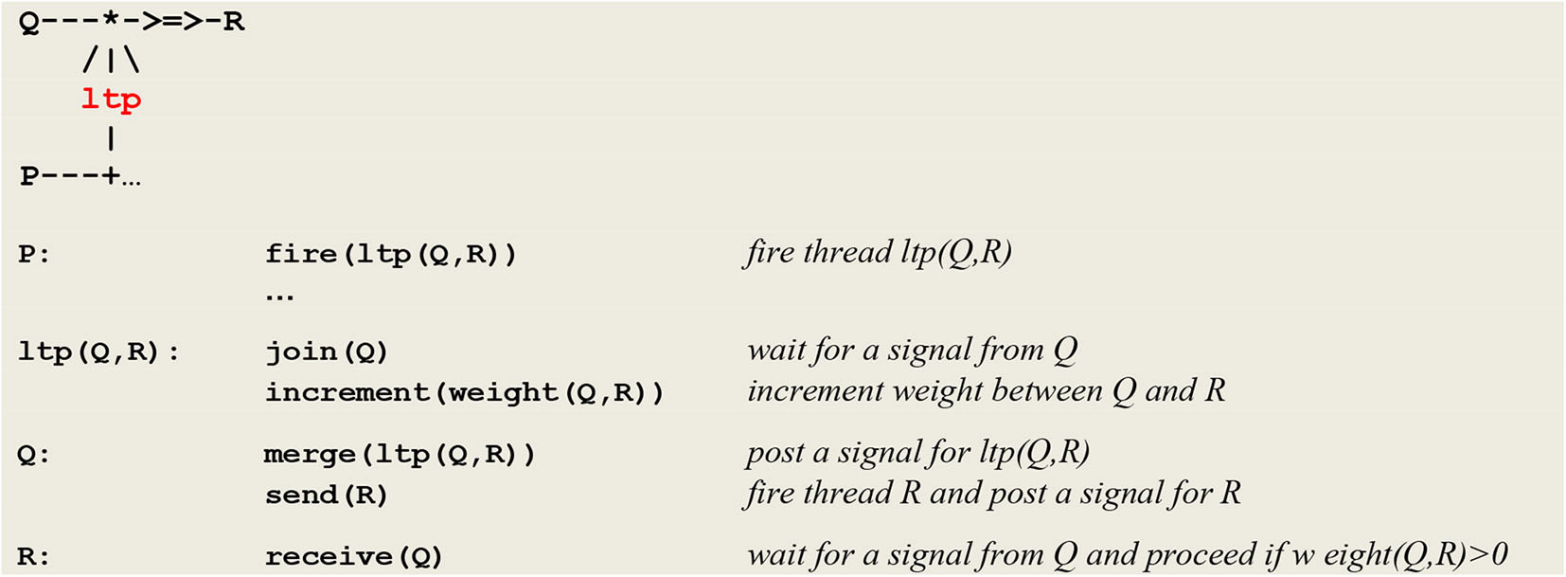
Micro-circuit and communication protocol for *ltp*. In order to detect the coincidence of **P** and **Q**, thread **P** fires an **ltp** thread that in turn calls on a **join** procedure to wait for a signal from thread **Q**. In parallel, thread **Q** calls on procedure **merge** to post a signal for **ltp** and then executes a **send®** command to establish a link with thread **R**. After its synchronization with thread **Q**, thread **ltp** increments the weight between **Q** and **R** (NB This protocol will be at the heart of the developments to come and will allow for the learning of causality rules).

The generic microcircuit abstracting the mechanism of long term potentiation is reproduced in Figure 6 with its communication protocol.

## Results

This section first presents the goals and design of a thought experiment, followed in turn by the implementation details and results of various simulation runs. Finally, an extended Hebbian learning principle is induced from a summary of these results.

### Goals of a Thought Experiment

By simulating observed behaviors supported by unknown cognitive processes, this work follows a behaviorist approach. Behaviorism was founded on the idea that the minds of humans and non-human animals alike have to be considered as black boxes i.e., as systems for which one does not postulate anything about the processes that control them, behaviors being then be defined solely by the input-output relations between sensations and actions. Behaviorist studies eventually culminated in the throughout exploration of operant conditioning (Skinner, 1953). In contrast, according to cognitivist views, behaviors are driven by mental states, which are themselves defined in brain internal structures.

In order to reconcile these two approaches, they should be both grounded in a common abstract biological substrate, leading to address the question: which neural structure could possibly drive an observed behavior and thus reveal its underlying cognitive processes? Behavioral rules can be considered as synthetic ways of expressing observed behaviors i.e., specific input/output relations, thus allowing for rephrasing the question as: “which neural structure could possibly be associated with a given behavioral rule?”

### Design of a Thought Experiment

**Figure 7:**
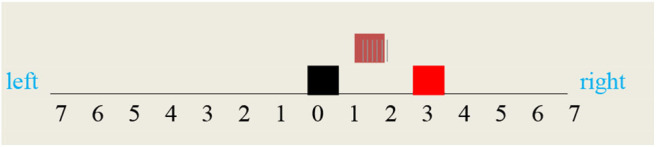
Experiment configuration. The **vehicle** is at **home**, smoke at right, and a fire at right(3).

In order to simulate a “thought experiment,” let us consider a vehicle that can move along a finite bidirectional track with discrete coordinates **F(X),** where **F****=**right or left and **X=1,..,7**. The coordinates _(0), where **F** is undefined, correspond to the vehicle home station. Let us further suppose that this vehicle is equipped with a forward and backward gear that allows him to move step by step in respectively the right and left direction, each step corresponding to moving from one coordinate to the next. In what follows, these two modalities will be systematically distinguished by their b and green color. Let us finally assume that this vehicle can perceive and/or access (see Figure 7)

- his current position
- a fire at his current position
- the smoke of a fire in a given direction.

**Figure 8:**
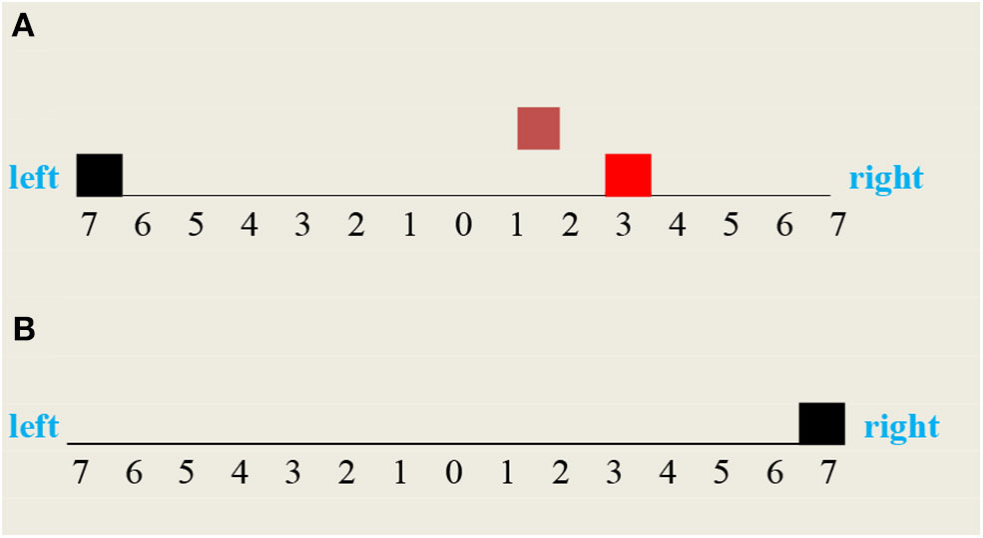
Vehicle two possible moves: **(A)** the **vehicle** moved backward to the left and missed the fire, **(B)** the **vehicle** moved forward to the right and cleared the fire.

The vehicle will be first programmed so that, upon perception of a smoke, it randomly selects its forward or backward gear and, starting from his home station, goes searching for the fire into the right or left direction. Regardless of having hit the fire (in which case it will **clear** it) or not, the vehicle then continues moving up to the track limit position and **stop** (Figure 8). The vehicle will eventually turn his smoke detector off, shift his gear and return to his home station, this last part not being reported in the simulation.

### Implementation of a Random Detector

**Figure 9:**
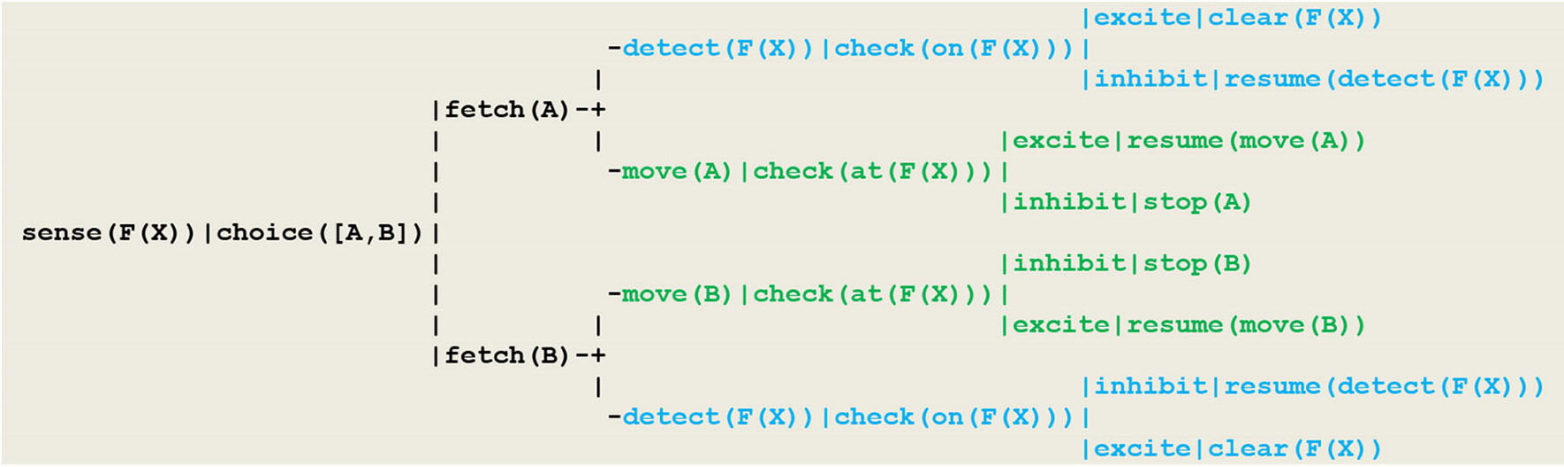
Circuit implementing a random detector. In this circuit, (1) detecting the smoke of a fire at location **F(X)** is implemented by the thread **sense(F(X))**, (2) the random choice between two gears **A** or **B** follows from the execution of the virtual machine instruction **choice([A,B])**that produces an internal *fetch* stimulus, (3) detecting the fire proceeds through the thread detect(F(X)), with the execution of the virtual machine instruction check(on(F(X))) producing an excite internal stimulus if the vehicle did hit the fire (in which case he will clear the fire) or an inhibit internal stimulus (in which case he will resume detecting) (4) moving is achieved through the move(A) or move(B) threads; depending on whether or not the current vehicle position F(X) is defined, the execution of the virtual machine instruction **check(at(F(X)))** similarly produces an internal excite (in which case he will resume moving) or an inhibit stimulus indicating that he has reached the track limit and must stop.

Using the formalism recalled above, this behavior can be driven by the circuit given in Figure 9, which in this case takes the form of a wired (i.e., non-plastic) random *decision tree*.

As an essential characteristic, these specifications do not use the equivalence relation which actually exists among the two basic modalities represented in blue and green i.e., the fact that in order to go right or left, one has to select respectively the forward and backward gear. For the simulation to be consistent however, the detect and move threads must be implemented such that whenever the vehicle has reached the fire location, both tests check(on(F(X))) and check(at(F(X))), although they refer to different modalities (i.e., being on the fire at a given location on the track), will provide an excite stimulus, thus signaling the *synchronization* of these two perceptions with respect to a global clock.

### Simulation Runs of a Random Detector

**Figure 10:**
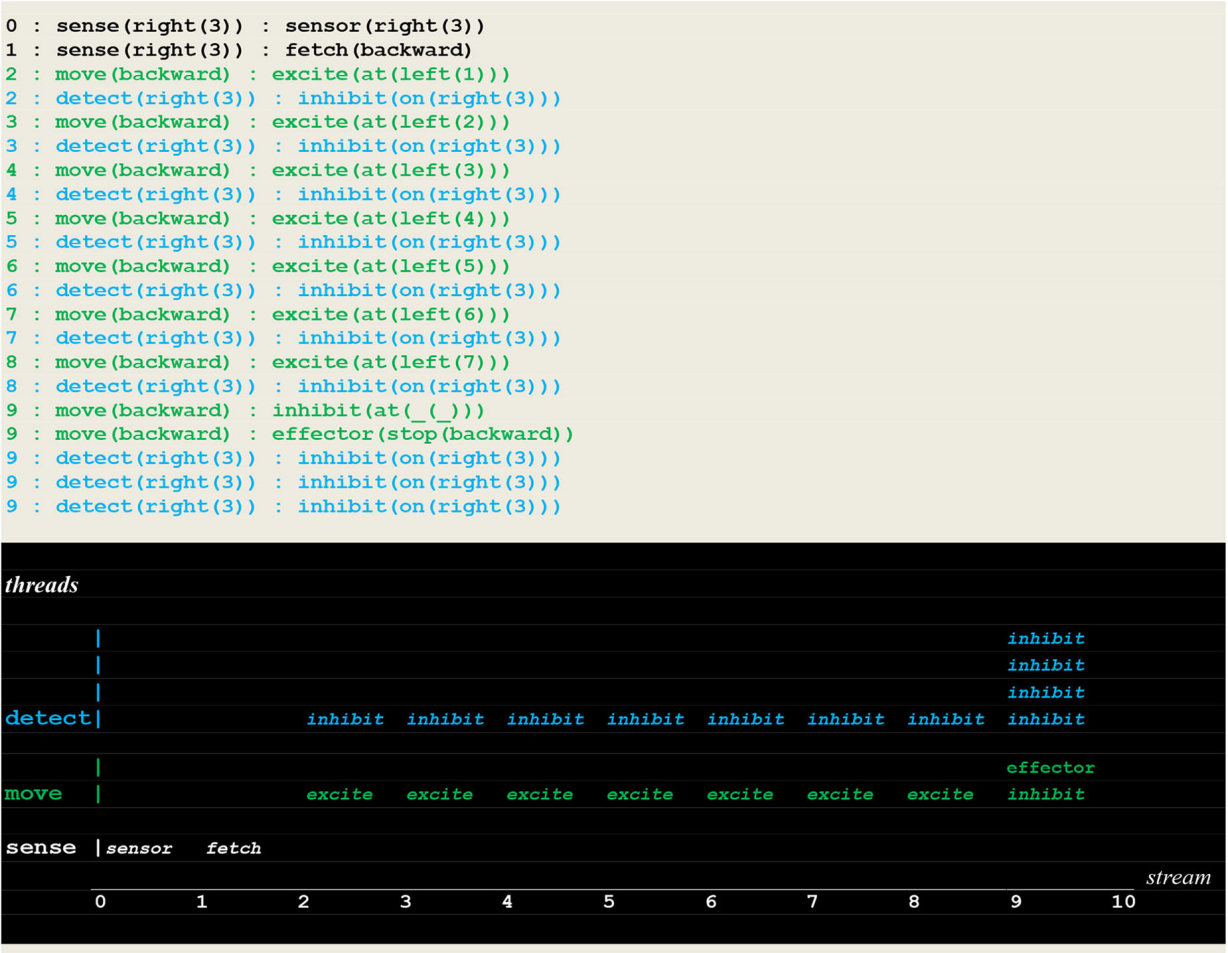
Execution trace of the vehicle randomly moving in the wrong direction. Following an incorrect **fetch(backward**) random choice, the detect and move concurrently produced a series of inhibit and excite stimuli until a single inhibit produced by the move thread at time 9 (signaling that the vehicle had reached the track limit) forced him to stop. At the same time, the detect threads went on producing inhibit stimuli (signaling that the fire had not been detected, and the detector not yet turned off).

**Figure 11:**
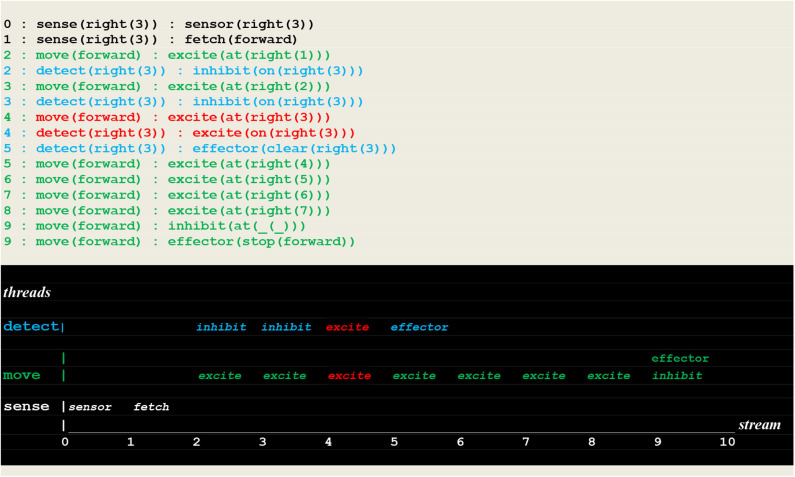
Execution trace of the vehicle randomly moving into the correct direction. Following a correct **fetch(forward)** random choice, two simultaneous excite internal stimuli indicate that two synchronized perceptions occurred, namely the detection of a fire at the vehicle's position. Subsequently the fire was cleared.

The execution traces of two simulation runs, where the vehicle first randomly moved into the left (Figure 10) and then into the right direction (Figure 11) are given below. As defined in section Extended Virtual Machine Definition, **these traces report synchronized events associating a stream sequence number I, a thread and a stimulus**. In these traces, prefixes 0:, 1:, 2:, 2:,.. are stream's sequence numbers ***I*** akin to a global time series. These sequence number correspond to successive move steps. Diagrams appearing below the traces have been constructed by hand from the actual simulation results in order to provide a more intuitive representation. In these diagrams, the global time is reported on the *stream* axis.

### Implementation of a Deterministic Detector

**Figure 12:**
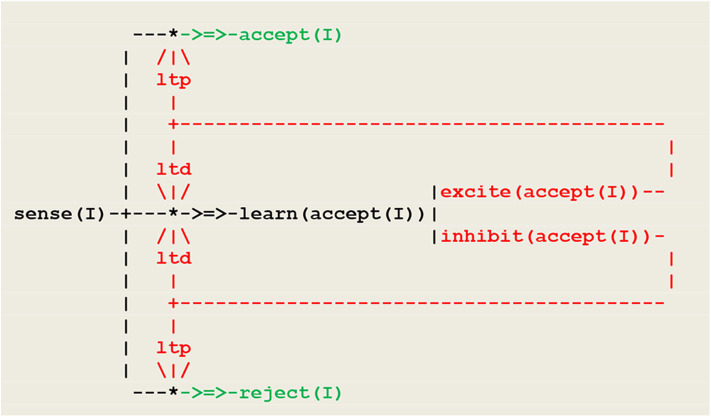
Neural circuit implementing a simple form operant conditioning. At the beginning, the pathway from **sense** to **learn** is open, while the pathways to accept and reject are closed. The **learn** thread discriminates between positive and negative external stimuli, and thus functions as a fork directing synaptic plasticity. Synaptic plasticity is expressed through ltp/ltd threads and eventually leads to open the path to either accept or reject, and close the path to **learn**.

In order to allow for the vehicle to adopt a deterministic behavior based on the correct association of the two basic modalities, synaptic plasticity will be introduced into the circuit of Figure 9, thus turning it into a virtual neural circuit implementing a form of *operant conditioning*. Operant conditioning is an associative learning process through which the strength of a behavior is modified by reward or punishment i.e., a case of reinforcement learning by positive or negative stimuli. In its simplest form, the operant conditioning response to a single perception is to generate either an excite or inhibit internal stimulus that in turn triggers an accept or reject action, as show in Figure 12.

**Figure 13:**
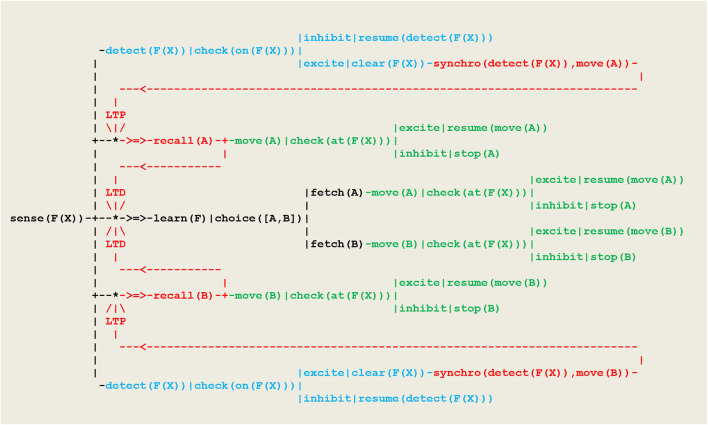
Circuit implementing a deterministic behavior. Let **Y** represent **A** or **B**. The ltp/ltd threads are activated via recall(Y) threads by synchronization threads synchro(detect(F(X)),move(Y)), which themselves fire whenever the tests check(on(F(X)))in detect(F(X))and check(at(F(X)))in move(Y) provide a simultaneous excite stimulus.

This procedure matches a fundamental principle in circuit neuroscience according to which, as a result of synaptic plasticity (expressed here through ltp/ltd threads), *inhibition* in neuronal networks allows in turn for *disinhibition* and constitutes a key mechanism for learning (Letzkus et al., 2015). The schema of Figure 12 will now be introduced in Figure 9 so that the ltp/ltd threads will be triggered by the synchronized multimodal perceptions of on(F(X)) and at(F(X)). The resulting neural circuit is given in Figure 13.

### Simulation Runs of a Deterministic Detector

Two learning cases will be distinguished depending on whether the vehicle learns one move at a time (i.e., learns and applies instantiated rules) or is able of generalized learning (i.e., learns and applies non instantiated rules):

1. the vehicle learns and applies instantiated rules e.g.,
  - if detect(right(3) then move(forward)
  - if detect(right(2) then move(forward), etc.
2. the vehicle learns and applies general rules i.e.,
  - if detect(right(_)) then move(forward)
  - if detect(left(_)) then move(backward)

#### Learning Single Moves

**Figure 14:**
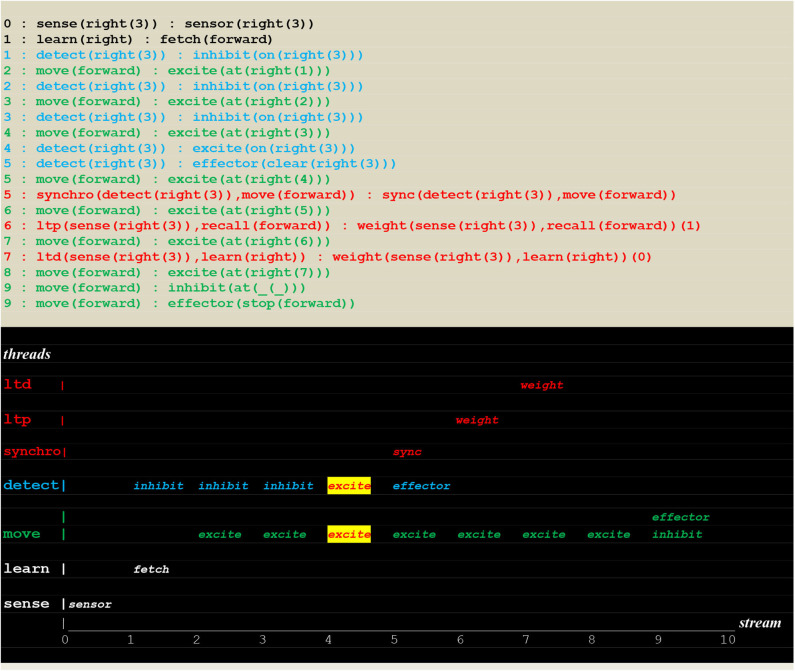
Execution trace of learning a single move. Facing an open path to **learn**, a random choice **learn(right):fetch(forward)** successfully led to find the fire at position **right(3)**, activating a synchro thread that initiated ltp/ltd threads (see Figure 5 for the communication protocol of ltp/ltd), thus inducing synaptic weight changes into instantiated links and leading in turn to both open the path to (sense(right(3)),recall(forward)) and close the path to (sense(right(3)),learn(right)).

A first example of a run performed to learn an instantiated rule with the circuit of Figure 13 is given in Figure 14.

**Figure 15:**
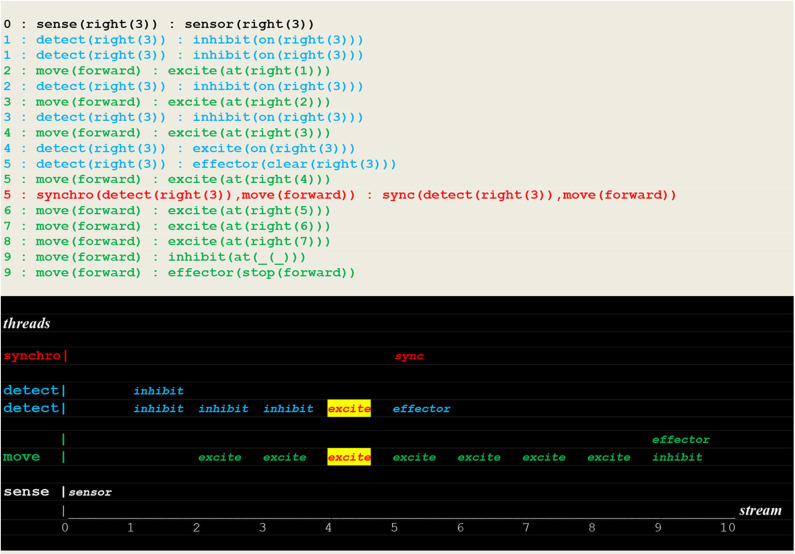
Execution trace of a repeated move. Facing a closed path to **learn**, the vehicle did proceed without **fetch** stimulus (but with a repeated inhibit at time 1), and went through the open path of recall to detect a fire at the same position as before.

The run reproduced in Figure 15 shows a successful deterministic run that just followed the learning of an instantiated move.

**Figure 16:**
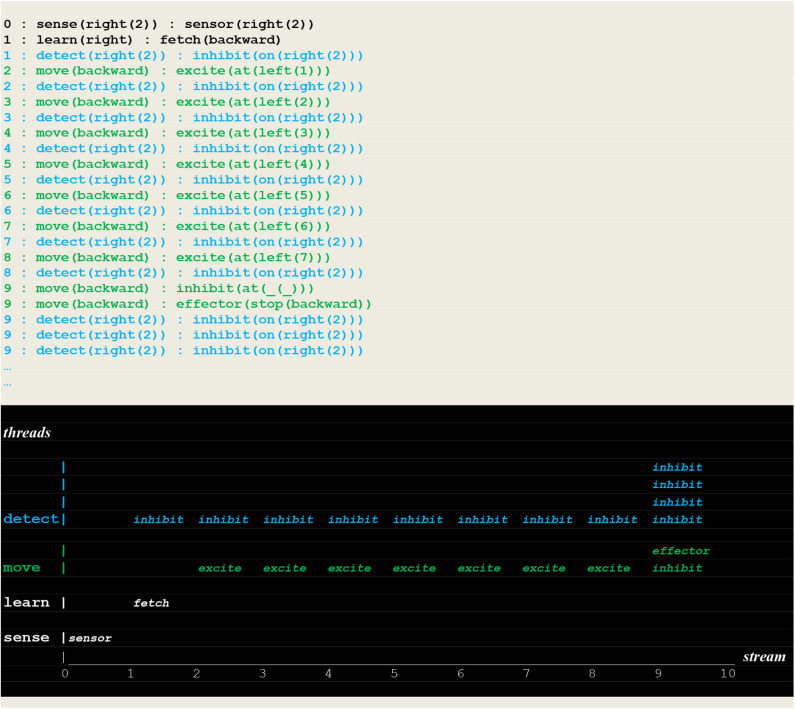
Execution trace of failing to detect a new fire: Facing an open path to **learn**, the vehicle made a random choice **learn(right):fetch(backward)** i.e., an incorrect choice that did not lead him to detect the fire.

In contrast, the run reported in Figure 16, which directly followed the two previous ones, shows a failure to detect a new fire located in the same direction but at a different position.

#### Learning Causality Rules

**Figure 17:**
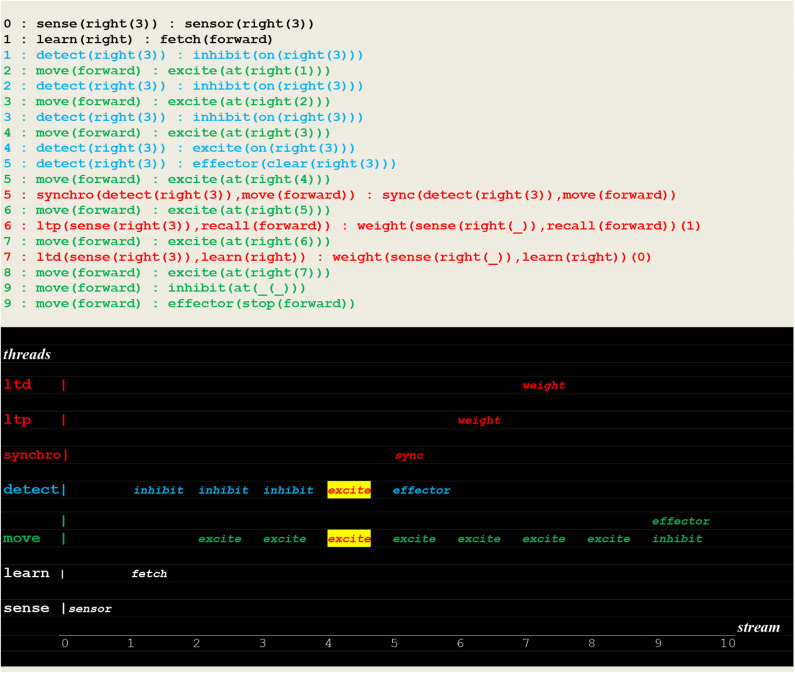
Execution trace of learning a non instantiated rule. This run is similar to the one presented in Figure 14, with the only difference being the weight changes applied to non instantiated links i.e., (sense(right(_)),recall(forward)) and (sense(right(_)),learn(right)).

The execution traces of Figure 17 demonstrate the learning of the rule “if detect(right(_)) then move(forward).” This was achieved by applying weight changes to non instantiated links.

**Figure 18:**
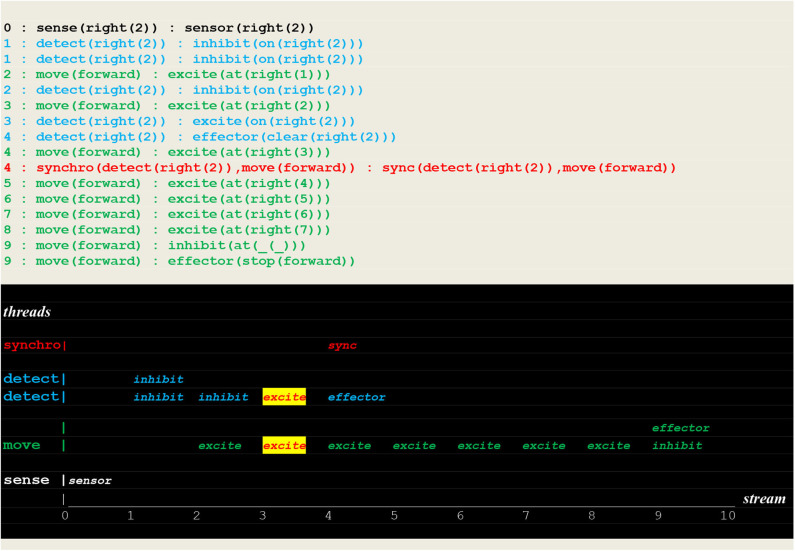
Execution trace of applying a rule. This run is similar to the one of Figure 15, but with a fire at different location in the same direction.

Finally, the run in Figure 18 shows a successful search for a fire at a different location in the same direction without going through learning i.e., by applying a learned rule.

### Results Assessment

In summary, the results of the work reported above are both methodological (i.e. provide a new simulation framework under the form of a virtual machine) and theoretical (i.e., propose an extended principle of hebbian learning that can be applied to learn deterministic behaviors).

On the methodological side, one must clearly distinguish between the implementation of the virtual machine itself, which is a computer program like any other brain simulator, and the brain processes it simulates, which are assembled from virtual microcircuits implementing synaptic plasticity. This virtual machine offers an interface situated at a meso-scale level between neural circuits and cognitive processes. This intermediate level is not claimed to “exist,” but is proposed as a way to delineate successive levels of complexity and to achieve a kind of independence and/or modularity between these levels.

On the theoretical side, the goal of this study was to explore synchronous vs. asynchronous processes associated with percepts and/or actions of different modalities. As evidence from the virtual neural circuit of Figure 13 that served as a basis to run simulations, we observe that:

- the ltp/ltd threads expressing synaptic plasticity and allowing in turn for inhibition/disinhibition processes are activated by synchronization threads synchro(detect(F(X)),move(Y))
- these synchronization threads themselves are triggered by simultaneous excite stimuli signaling the synchronized multimodal perceptions of on(F(X))and at(F(X))
- the learning of single equivalences under the form of instantiated rules e.g., “if detect(right(3) then move(forward)” follow from synaptic weight changes that are applied to instantiated links
- the learning of an equivalence relation given under the form of general rules e.g., “ if detect(right(_) then move(forward)” similarly follow from synaptic weight changes that are applied to non instantiated links.

This leads to induce an extended Hebb principle formulated as follows:

Extended Hebbian learning principle

Within a stream of multimodal perceptions supported by asynchronous threads, simultaneous internal stimuli trigger inhibition and disinhibition processes driven by synaptic plasticity; these processes allow for learning equivalence relations among different modalities; these relations actually represent causality rules and lead in turn to define deterministic behaviors.

## Discussion

This discussion will extend in three directions i.e., the relevance of the proposed formalism to bio-inspired robotics, the use of simulated thoughts experiments as a part of the scientific method, and finally limitations, comparisons, and possible future work.

### Relevance of the Proposed Formalism

The two main formalisms used today in computational neuroscience follow from pioneering work dating at about the same time i.e., the work of McCulloch and Pitts (1943) defining abstract finite-state automata that implement a threshold logic and led to the development of *artificial neural networks*, on one hand, and the analytical treatment of Hodgkin and Huxley (1952) simulating the electrical processes surrounding neurons and forming the basis of neural simulators, on the other. These two formalisms do not address the behavioral learning dimension of cognition. In his assessment of this situation, Poggio (2012) argues that in order to discover the representations used by the brain, one needs to understand “*how* an individual organism learns and evolves them from experience of the natural world,” and that “learning algorithms and their a priori assumptions are deeper and more useful than a description of the details of *what* is actually learned.” On their side, van der Velde and de Kamps (2015) note that “cognitive processes are executed in connection structures that connect sensory circuits to motors circuits” i.e, sensations with actions. Therefore, they add, “we need the description of a mechanism that shows how the information (*synchrony* of activation in this case) can be used by the brain.” This should be accomplished through a “*middle-out*” approach identifying plausible structures linking biology and cognition (Mulder et al., 2014; Frank, 2015).

As introduced in software engineering, the concept of a virtual *machine* allows for a strict delineation of successive levels of complexity. In the present context, by providing an interface situated at a meso-scale level between abstracted micro-circuits representing synaptic plasticity, on one hand, and that of the emergence of behaviors, on the other, this tool has been used to simulate yet unknown processes of cognition independently of their underlying neurological grounding. As an example, formal studies of consciousness focus on the so-called “neural correlates of consciousness” i.e., on the “search for the minimal neural mechanisms sufficient for any one specific conscious percept” (Koch et al., 2016). As evidenced from our simulation results, the random detector implemented in this study to drive an autonomous vehicle does not incorporate the equivalence between the right/left and the forward/backward modalities. Nor does it allow for learning it. Consequently, the vehicle cannot change his behavior. In contrast, the detector endowed with synaptic plasticity and relying on an extended Hebb learning principle learns and adopts a deterministic behavior corresponding to the application of a causality rule, just as a conscious human driver would do.

### Using Simulated Thought Experiments

Arguments and developments relying on thought experiments are sometimes dismissed as lacking any scientific value. One has to distinguish however between thought experiments which remain at the level of discourse from those actually implemented through a simulation. This later kind, as used here, truly allows for trying to reproduce the reality of nature and possibly unfold some causality rules. More precisely, and in accordance with the requirements of the scientific method, they do allow for formalizing hypothesis involving a theory and leading to predictions, and then confront these predictions with experimental observations. Contrary to some thoughts experiments proposed under the form of mere abstract constructions that could never be materialized (e.g., such as the so-called “Schrödinger cat” illustrating concepts in quantum physics), the thought experiment proposed here could be attempted in its corresponding concrete form. Thought experiments designed and implemented as virtual machines could thus eventually provide formal specifications for neuro-robotics developments.

### Limitations, Comparisons, and Further Directions

As indicated in the Material and methods section, the virtual machine considered in this study was implemented in Prolog i.e., a language that can be coupled with other programming environments, but is not appropriate for controlling real-time systems. Interfacing this virtual machine with an actual vehicle might thus not be an easy task. It is proposed as a challenge to the neuro-robotic community.

To contrast this new approach with the more traditional one, let us point out to the recent contribution of Zeng et al. (2020). In this work, anatomically distinct brain areas, as identified by previously published experimental research, are first simulated through a *leaky integrate-and-fire* (LIF) neuron model (Gerstner and Kistler, 2002), and then connected to interact through control methods tailored to perform specific tasks, thus reconstructing parts of a functional brain. The LIF neuron model was obtained by trimming the original Hodgkin and Huxley differential equations, thus resulting in a computationally tractable model that, contrary to the more complex ones that have culminated in recent years (see e.g., Markram et al., 2015), allows for a feasible simulation of a functional brain.

Reconstructing a functional brain as it exists today somehow constitutes an attempt to reproduce the results of millions of years of evolution. This evolution process started with simple unimodal insect and mollusk brains, gradually adding specialized brain components endowed with more and more complex multimodal mechanisms, to eventually end up with the human brain. In contrast, as emphasized throughout this paper, the aim of the present work was to try and identify a general learning principle that could be applied to simulate from scratch the capabilities of an inherently multimodal brain in isolation from its underlying biological substrate.

In order to discuss the continuity between animals and humans from a developmental point of view and possibly extent it into vehicles behavior, let us quote (Darwin, 1871): “*the difference in mind between man and the higher animals, great as it is, certainly is one of degree and not of kind*.” Following the modeling of the first three levels of animal awareness reported in the Introduction, the same approach has been applied to simulate simple forms of both *meta-cognition* (namely *memory awareness*, which provides subjects with the capacity to make “judgments” about the quality of their memory e.g., to discriminate between strong and weak memories) and *analogical reasoning* (in this case, by applying *inference schemas* that allow for discovering structural and/or functional similarities between sets of objects e.g., finding out that “*large blue triangle*” is to “*small blue triangle*” as “*large yellow crescent*” is to “*small yellow crescent*”). These results (Bonzon, 2017b) indicate first that memory awareness can be reduced to successive layers of associative processes driven by noisy transmissions, which is quite compatible with the hypothesis of an overall continuity between human and nonhuman minds. Secondly whereas analogical inference schemas involving one kind of transfer (i.e., the transfer of either a *property* or a *subject*) correspond to cognitive abilities observed in animals, schemas involving a combination of transfers correspond to abilities presumably enjoyed by humans only. This gap however could be ignored when building artifacts with extended capabilities such as those just discussed.

More generally, further work should try and integrate sensory inputs from more realistic experiments situated in a two- or three-dimensional space. These extensions might eventually turn the original virtual machine into a kind of programmable Braitenberg vehicle with wired connections replaced by cognitive software.

## Data Availability Statement

The original contributions presented in the study are included in the article, further inquiries can be directed to the corresponding author.

## Author Contributions

The author confirms being the sole contributor of this work and has approved it for publication.

## Conflict of Interest

The author declares that the research was conducted in the absence of any commercial or financial relationships that could be construed as a potential conflict of interest.
